# Asynchronous, digital teaching in times of COVID-19: a teaching example from general practice

**DOI:** 10.3205/zma001391

**Published:** 2020-12-03

**Authors:** Piet van der Keylen, Nikoletta Lippert, Raphael Kunisch, Thomas Kühlein, Marco Roos

**Affiliations:** 1 Friedrich-Alexander-Universität Erlangen-Nürnberg, Institute of General Practice, Erlangen, Germany

**Keywords:** digital teaching, COVID-19, general practice, inverted-classroom, blended-learning

## Abstract

**Background: **The SARS-CoV-2 pandemic had a strong impact on academic teaching and could change it sustainably. Ad hoc digitization of teaching had to be carried out.

**General practice teaching situation**: Education in general practice at the Friedrich-Alexander-Universität Erlangen-Nürnberg (FAU) offers, in addition to the main lecture, various elective courses, clinical traineeships, internship as well as the elective part in the final practical year. The main lecture and one clinical elective course were offered digitally in the summer term 2020.

**Digital methods:** In the main lecture, an adapted inverted-classroom concept was used. Podcasts and audio annotated videos were provided. Teaching materials were reflected via a weekly, 1hr video consultation and in a forum. An asynchronous learning module was developed for the elective course *“Smart Decision-making in Clinical Practice”*. Each module consisted of course preparation, podcasts and follow-ups as well as a supervised forum.

**Results: **The main lecture (response rate *n*=115/170; 67.6%) was rated *“very good”* on average. The same applies to the commented videos. The forum, reflective video consultation and teaching materials were rated *“good”* on average. The predominantly desired forms of presence were* “Focus on virtual with in-depth presence phases”* (*n*=54) and *“Focus on presence phases, virtual support only” *(*n*=37).

**Discussion and implications: **The digital restructuring enables students to work on the course contents independently. This requires self-regulation strategies, which in future shall be taught through portfolio work. The teaching focus shifts from a passive teaching format to an interactive one. First evaluation results showed a very good acceptance by the students.

## Zusammenfassung

**Hintergrund: **Die SARS-CoV-2-Pandemie wirkte sich stark auf den akademischen Lehrbetrieb aus und könnte diesen nachhaltig verändern. Ad hoc musste eine Digitalisierung des Lehrbetriebs durchgeführt werden.

**Allgemeinmedizinische Lehrsituation:** Die allgemeinmedizinische Lehre an der Friedrich-Alexander-Universität Erlangen-Nürnberg bietet neben der Hauptvorlesung diverse Wahlfächer, Famulaturen, Blockpraktika sowie das Wahltertial im praktischen Jahr an. Die Hauptvorlesung und ein klinisches Wahlfach wurden im Sommersemester 2020 digital angeboten.

**Digitale Methoden: **Die Hauptvorlesung bediente sich eines adaptierten inverted-classroom Konzepts. Podcasts und audio-kommentierte Videos wurden bereitgestellt. Lehrmaterialen wurden per Videokonferenz, sowie in einem Forum reflektiert. Für das Wahlfach *„Kluge Entscheidungen im klinischen Alltag“* wurde ein asynchrones Lernmodul entwickelt. Jeder Themenblock bestand aus einer Kursvorbereitung, Podcasts und Nachbereitungen sowie einem betreuten Forum.

**Ergebnisse: **Die Hauptvorlesung (Rücklaufquote *N*=115/170; 67,6 %) wurde durchschnittlich mit *„sehr gut“* bewertet. Ebenso die kommentierten Videos. Das Forum, die Videosprechstunde und Lehrmaterialien bekamen durchschnittlich die Note *„gut“*. Die überwiegend gewünschten Präsenzformen waren *„Schwerpunkt digital mit vertiefenden Präsenzphasen“* (*N*=54) und *„Schwerpunkt Präsenz, nur digital unterstützend“* (*N*=37).

**Diskussion und Implikationen: **Die Studierenden können durch die digitale Umstrukturierung eigenverantwortlich Lerninhalte erarbeiten. Dafür sind Selbstregluationsstrategien vonnöten, welche mittels Portfolioarbeit zukünftig vermittelt werden sollen. Der Lehrfokus verschiebt sich von einem passiven Lehrformat hin zu einem interaktiven. Erste Evaluationsergebnisse verdeutlichten eine sehr gute Akzeptanz seitens der Studierenden.

## 1. Background

In the wake of the SARS-CoV-2 pandemic, measures were initiated in Bavaria which could change academic teaching sustainably [1]. The challenge lay in the short-term transformation of established courses into a digital format. Although the Master Plan 2020, irrespective of the current situation [2], points to the urgency of digitizing medical teaching, it was the SARS-CoV-2 pandemic that created the necessary pressure to digitize teaching and implement knowledge transfer in a creative and proactive way for participants. The following report is intended to show how general practice teaching can work digitally and asynchronously in the clinical section of the study program, thus going didactically beyond the conventional frontal classroom teaching. Interactive learning activities according to the *“ICAP Framework”* can be supported by the *“Reflective Video Consultation”* and an *“Online Forum” *[3]. These enable the experience of autonomy and competence as well as relatedness, which according to self-determination theory have a positive influence on learning motivation and performance [4]. 

## 2. General practice teaching situation

**Figure 1:**
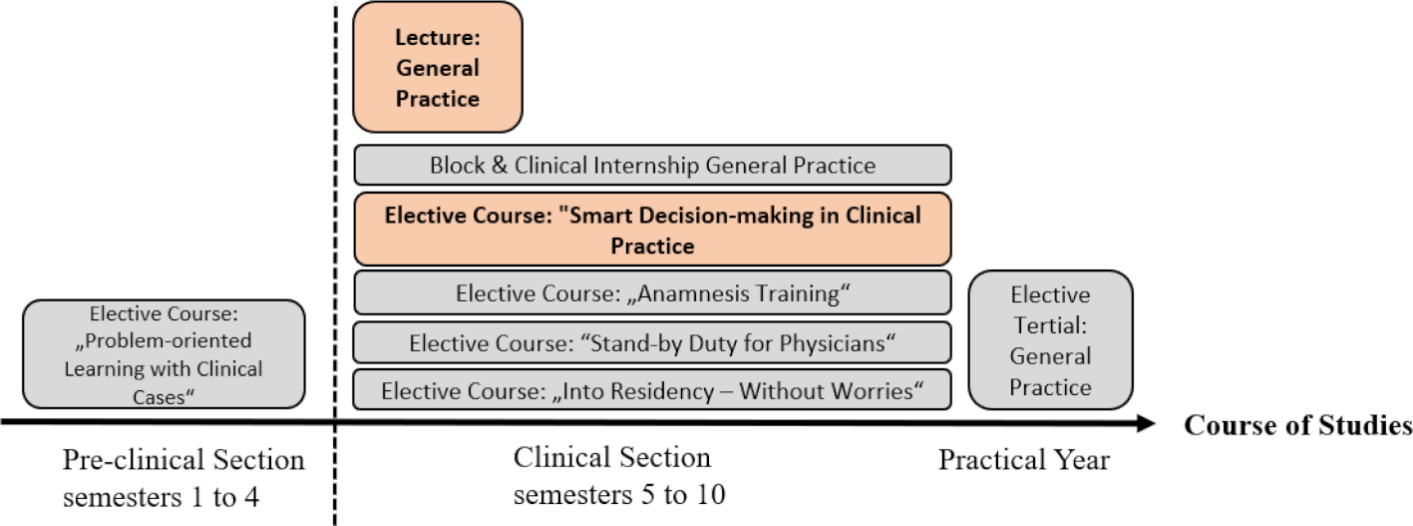
Presentation lecture and elective courses coursed at the Friedrich-Alexander-Universität Erlangen-Nürnberg (FAU) for the summer term 2020. Courses which have been modified to a solely digital teaching format within the context of the digital semester are highlighted in color (orange).

At the Friedrich-Alexander-Universität Erlangen-Nürnberg (FAU), general practice is anchored in the 1^st^ clinical semester as a lecture with two semester hours per week (SWS). The internship is to be completed from the 5^th^ to the 10^th^ semester. (Pre-)clinical elective courses are offered. The practical elective part can be completed in the final practical year (see figure 1 (Fig. 1)). In the summer term 2020, the *“Lecture General Practice”* and the clinical elective course *“Smart Decision-making in Clinical Practice”* were offered digitally. Other elective courses could not be offered digitally due to the highly practice-oriented interactive setting (e.g., small groups, role-play), or – after suspension – were offered again in analogue form (clinical traineeship, internship, elective part).

## 3. Digital methods

### 3.1. Lecture General Practice

The lecture was transformed into a solely digital teaching format. The 90-minute lectures were offered as video podcasts. Each lecture was divided into smaller thematic sections (*“Segmenting Principle”*) [5] and made available as audio annotated videos via ILIAS, a learning platform [https://www.studon.fau.de/]. In-depth assignments for self-study and further materials were offered to counteract the possible passivity by reduced interaction. The contents were made available one week before the actual *“presence date”*. At this meeting, a reflective video consultation with practical examples took place on WebEx (Cisco Systems, [https://www.webex.com/]). A forum on the learning platform, supervised by the lecturers, encouraged additional interaction. This resulted in a variant of an inverted-classroom concept with the possibility of reflexive learning in an asynchronous environment and at an individual pace [6]. To promote digital learning, the formats were held in common language, and the video lesson was conducted by two lecturers in dialogue with each other and with the students, in accordance with Mayer's *“Personalization Principle”* [7]. At the end of the lecture, students evaluated their learning experience. This included self-developed items for the evaluation of didactic methods, satisfaction and the desired form of presence. Items were answered, following the school grading principle (1 *“very good”* through 6 *“deficient”*). A free text answer (open wishes/criticism) was also possible.

#### 3.2. Elective "Wise Decision-Making in Clinical Practice”

The clinical elective course was initially established as a hybrid event (presence-digital, 2 SWS). In the summer term 2020 it was further developed as an online learning module (*ILIAS*, [https://www.studon.fau.de/]) and interlocked with the lecture thematically and didactically. For each thematic section, there was an activating preparation, supplemented by podcast contributions of the lecturers. A course follow-up intensified the content in self-study by means of exercises or control questions and thus informed students about the learning progress. A forum supervised by lecturers and student tutors enabled asynchronous interaction.

## 4. Results

**Figure 2:**
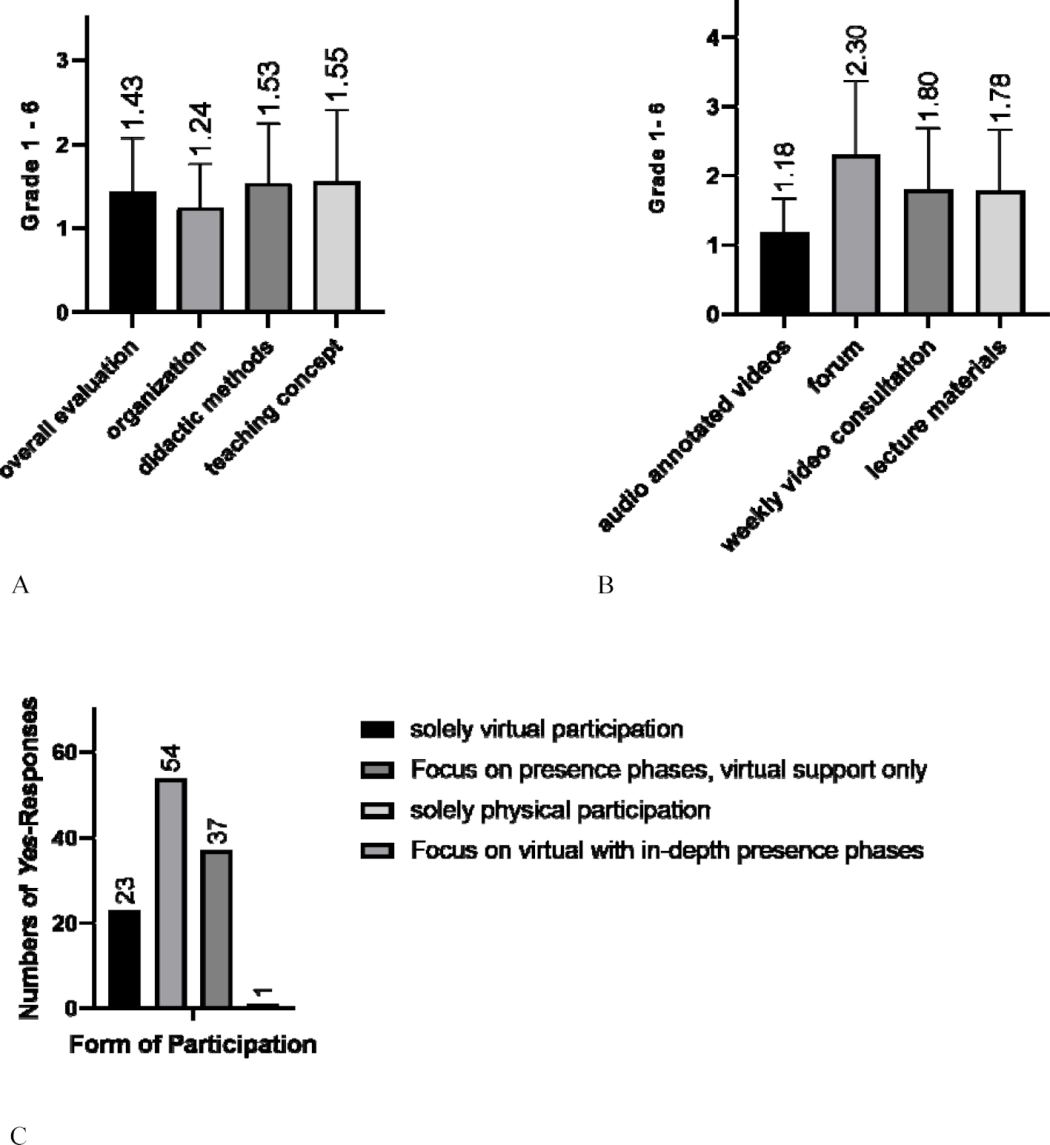
A=General evaluation of the lecture on general practice in the summer semester 2020; e.g. *“How do you rate [overall] the digital lecture series for general practice?”*; German school grading system (n=115). B=Evaluation of the digital elements used; *“How suitable do you consider the used elements to support your self-study?”*; German school grading system (n=115). C=Form of participation desired by students; dropdown question on the type of presence desired (n=115). *Graphs and values were calculated and created using *GraphPad Prism* (V.8.3.0) [https://www.graphpad.com/guides/prism/7/user-guide/citing_graphpad_prism.htm].

Evaluation results – assessment according to German school grades – of the lecture (response rate *n*=115/170; 67.6%, see figure 2 (Fig. 2)) showed a high level of acceptance with an average grade of *“very good”*. The same applies to the commented videos. Forum, reflective video consultation and teaching materials received an average grade of *“good”*. The most desired teaching formats were* “Focus on virtual with in-depth presence phases”* (*n*=54) and *“Focus on presence phases, virtual support only”* (*n*=37). In the free-text responses, the* “very good organization”* and *“didactic method”* were highlighted in comparison to other clinical subjects. The evaluation of the elective course has not yet been completed and will be published elsewhere.

## 5. Discussion and implications

1. Asynchronous teaching enables students to work independently at their individual learning pace, regardless of time and place. Nevertheless, personal contact must be possible at all times. In addition to content and method, students appreciate reliable structure, organization and support in a virtual environment.
2. Students need self-regulation strategies to ensure that online courses do not become more boring and less enjoyable than in class [8]. Self-regulation can be encouraged, for example, through portfolio work [9].
3. Asynchronous, *digital blended-learning/inverted-classroom* concepts can strengthen clinical teaching (e.g. general practice) [10], [11]. The focus is shifting from passive teaching of content to interactive content handling.
4. Evaluation differences between videos and the forum may result from a different level of activity (passive vs. interactive) [3].
5. A further development of the evaluation is planned.

## Funding

The described elective is a course for the introduction to evidence-based medicine. It is recognized as a continuing education course (*DNEbM e.V.*) and is funded by the *German Federal Ministry of Education and Research* (*“QuiS II No. 01PL17017”*).

## Authorship

PK and NL share first authorship and were responsible for manuscript preparation, data collection and analysis as well as the development of the evaluation. RK and TK revised the manuscript. MR developed the evaluation and, together with PK, was responsible for the development of the teaching idea. MR revised the manuscript, data collection and data presentation.

The present work was performed in (partial) fulfillment of the requirements for obtaining the degree “Dr. rer. biol. hum.” for NL.

## Acknowledgements

The authors thank the student tutors for their support. The authors would like to thank the Medical Faculty of the FAU Erlangen-Nuremberg and the Institute for Learning Innovation of the FAU Erlangen-Nuremberg for their constructive support.

## Competing interests

The authors declare that they have no competing interests. 

## References

[1] Bayerisches Staatsministerium für Gesundheit und Pflege (2020). Coronavirus - Maßnahmen 2020.

[2] Bundesministerium für Bildung und Forschung (2017). Masterplan Medizinstudium 2020.

[3] Chi MT, Wylie R (2014). The ICAP framework: Linking cognitive engagement to active learning outcomes. Educ Psychol.

[4] Deci EL, Ryan RM (200011(4)). The "What" and "Why" of Goal Pursuits: Human Needs and the Self-Determination of Behavior. Psychol Inq.

[5] Clark R, Mayer R (2010). Applying the segmenting and pretraining principles: managing complexity by breaking a lesson into parts. E-Learning and the Science of Instruction: Proven Guidelines for Consumers and Designers of Multimedia.

[6] Tolks D, Schäfer C, Raupach T, Kruse L, Sarikas A, Gerhardt-Szép S, Klauer G, Lemos M, Fischer MR, Eichner B, Sostmann K, Hege I (2016). An introduction to the inverted/flipped classroom model in education and advanced training in medicine and in the healthcare professions. GMS J Med Educ.

[7] Mayer RE, Mayer RE (Cambridge University Press: 2005). Principles of multimedia learning based on social cues: Personalization, voice, and image principles. The Cambridge handbook of multimedia learning.

[8] Stephan M, Markus S, Gläser-Zikuda M (2019). Students' Achievement Emotions and Online Learning in Teacher Education. Front Educ.

[9] Gläser-Zikuda M, Fendler J, Noack J, Ziegelbauer S (2011). Fostering self-regulated learning with portfolios in schools and higher education. Orbis scholae.

[10] Engel B, Esser M, Bleckwenn M (2019). Piloting a blended-learning concept for integrating evidence-based medicine into the general practice clerkship. GMS J Med Educ.

[11] Northey G, Bucic T, Chylinski M, Govind R (2015). Increasing student engagement using asynchronous learning. J Market Educ.

